# Optimizing Prognostic Predictions in Liver Cancer with Machine Learning and Survival Analysis

**DOI:** 10.3390/e26090767

**Published:** 2024-09-07

**Authors:** Kaida Cai, Wenzhi Fu, Zhengyan Wang, Xiaofang Yang, Hanwen Liu, Ziyang Ji

**Affiliations:** 1 Department of Epidemiology and Biostatistics, School of Public Health, Southeast University, Nanjing 210009, China; 2 Department of Statistics and Actuarial Science, School of Mathematics, Southeast University, Nanjing 211189, China; 220241993@seu.edu.cn (W.F.); zhengyanwang@seu.edu.cn (Z.W.); xiaofangyang@seu.edu.cn (X.Y.); 220242066@seu.edu.cn (H.L.); 213200682@seu.edu.cn (Z.J.); 3 Key Laboratory of Environmental Medicine Engineering, Ministry of Education, School of Public Health, Southeast University, Nanjing 210009, China

**Keywords:** liver hepatocellular carcinoma, machine learning, feature selection, information gain, survival analysis

## Abstract

This study harnesses RNA sequencing data from the Cancer Genome Atlas to unearth pivotal genetic markers linked to the progression of liver hepatocellular carcinoma (LIHC), a major contributor to cancer-related deaths worldwide, characterized by a dire prognosis and limited treatment avenues. We employ advanced feature selection techniques, including sure independence screening (SIS) combined with the least absolute shrinkage and selection operator (Lasso), smoothly clipped absolute deviation (SCAD), information gain (IG), and permutation variable importance (VIMP) methods, to effectively navigate the challenges posed by ultra-high-dimensional data. Through these methods, we identify critical genes like MED8 as significant markers for LIHC. These markers are further analyzed using advanced survival analysis models, including the Cox proportional hazards model, survival tree, and random survival forests. Our findings reveal that SIS-Lasso demonstrates strong predictive accuracy, particularly in combination with the Cox proportional hazards model. However, when coupled with the random survival forests method, the SIS-VIMP approach achieves the highest overall performance. This comprehensive approach not only enhances the prediction of LIHC outcomes but also provides valuable insights into the genetic mechanisms underlying the disease, thereby paving the way for personalized treatment strategies and advancing the field of cancer genomics.

## 1. Introduction

Liver hepatocellular carcinoma (LIHC) is a major form of liver cancer and a significant contributor to cancer-related deaths globally [1,2]. Its high incidence and mortality rates are alarming, often attributed to underlying conditions such as liver cirrhosis, chronic hepatitis B virus, hepatitis C virus infections, alcoholic liver disease, and nonalcoholic fatty liver disease [3]. The prognosis for LIHC remains poor, with a five-year survival rate under 20%, primarily due to late-stage diagnoses. Current treatment options, including Sorafenib and immune checkpoint inhibitors like Nivolumab and Ipilimumab, offer some improvement in survival but are not sufficient for long-term management [4,5]. This highlights the urgent need for more effective therapeutic strategies. Understanding the genetic underpinnings of LIHC could pave the way for better prognostic markers and personalized treatments, which is the focus of our study.

The human genome contains numerous genes that can influence the development and progression of cancer. Identifying the key genes strongly associated with LIHC involves employing robust variable selection techniques to eliminate irrelevant genes. The ultra-high-dimensional nature of gene expression data, where the number of features significantly exceeds the sample size, presents a substantial challenge for traditional feature selection methods [6]. Therefore, it is essential to first utilize feature selection methods specifically designed for ultra-high-dimensional data to reduce the number of features below the sample size [6,7]. To address the challenges of ultra-high-dimensional data, Fan and Lv [6] introduced the sure independence screening (SIS) method, which effectively reduces the dimensionality of feature space, making it manageable for further analysis using traditional feature selection techniques. Following this, Fan et al. [7] extended these methods to the Cox proportional hazards model, providing a robust framework for high-dimensional feature selection in survival analysis. Additional significant contributions to the field include those of Zhao and Li [8], who developed a principled screening method for Cox models with ultra-high-dimensional covariates, and Zhu et al. [9], who proposed a model-free feature screening approach for ultra-high-dimensional data. After reducing the data dimension, penalized methods such as the least absolute shrinkage and selection operator (Lasso) and smoothly clipped absolute deviation (SCAD) are employed to continue the variable selection process, ensuring that only the most relevant variables are retained [10,11]. Also, information theory concepts like entropy can be instrumental in feature selection by quantifying the uncertainty and information gain of different features, thereby aiding in the dimensionality reduction process [12].

To develop personalized treatment strategies, it is necessary to perform survival analysis on the expression levels of key genes and the survival times of cancer patients, which can predict patient survival time and recurrence risk [13]. Survival analysis is a statistical method used to analyze the timing of endpoint events in study subjects and is widely applied in various fields to elucidate the relationships between factors and the occurrence of endpoint events under temporal changes [14]. The Cox proportional hazards regression is one of the most classical statistical methods in survival analysis [14]. However, traditional statistical methods like the Cox model have limitations, such as assuming proportional hazards and requiring linear relationships between features. Machine learning is widely applied in the field of survival analysis because it can extract valuable data information from massive, nonlinear, noisy, and heterogeneous datasets without needing any assumptions about the data distribution [15]. Gordon and Olshen [16] improved the methods of classification and regression trees, successfully applying tree-structured methods to the analysis of truncated survival data, thus enabling their widespread application in medical follow-up studies. Khan and Zubek [17] proposed a support vector regression for handling censored data in survival analysis. Ishwaran et al. [18] extended the random forest method to the domain of survival analysis, creating the random survival forests model. Random survival forests, being a data-driven learning algorithm that is entirely nonparametric, can automatically handle the nonlinear effects and interactions among features.

In recent years, advancements in high-throughput sequencing technologies have enabled researchers to explore the genetic underpinnings of complex diseases like liver hepatocellular carcinoma (LIHC) with unprecedented detail. Traditional survival analysis methods, such as the Cox proportional hazards model, have been extensively used to understand the factors influencing patient survival times [14]. However, these methods often struggle with high-dimensional datasets, where the number of predictors far exceeds the number of observations. In this study, we leverage the advancements of feature selection methods to analyze RNA sequencing data from the Cancer Genome Atlas database for LIHC. We first apply the sure independence screening (SIS) method to reduce the dimensionality of the dataset, followed by penalized feature selection methods using Lasso and SCAD, as well as the information gain method [7,12,19,20]. We also incorporate permutation variable importance (VIMP) from random survival forests (RSF) to further refine the feature selection process. Subsequently, we employ three survival analysis methods, including the Cox model, survival tree, and random survival forests, to analyze the data with the selected features. This approach allows us to compare the performance of traditional statistical methods with machine learning methods. Our findings demonstrate that the random survival forests method consistently achieves the highest predictive performance, highlighting its robustness in handling complex, high-dimensional data. Notably, our approach identifies critical genetic markers, such as MED8, which play significant roles in LIHC progression and offer potential as biomarkers for prognostic assessment. This integrated methodology not only improves the predictive accuracy of survival methods but also provides valuable insights into the genetic mechanisms underlying LIHC, contributing to the development of personalized treatment strategies and advancing the field of cancer genomics.

## 2. Methods

### 2.1. Data Source

In this study, we analyze RNA sequencing data and the accompanying clinical features for liver hepatocellular carcinoma (LIHC) from the Cancer Genome Atlas (TCGA) database, accessed through the Genomic Data Commons (GDC) portal. The dataset initially comprises 424 samples, each characterized by 60,616 gene expression features. To ensure data homogeneity conducive to robust survival analysis, we exclude 53 samples that are classified neither as primary tumor nor solid issue normal. Additionally, to preserve the integrity of the survival analysis, we remove any samples lacking a recorded survival status or displaying a survival time of zero. Simultaneously, gene features that exhibit zero expression across all retained samples are also excluded, aligning with best practices in data preprocessing for high-dimensional biological data. After these stringent selection criteria, the dataset includes 364 samples, encompassing 56,268 gene features.

**Figure 1:**
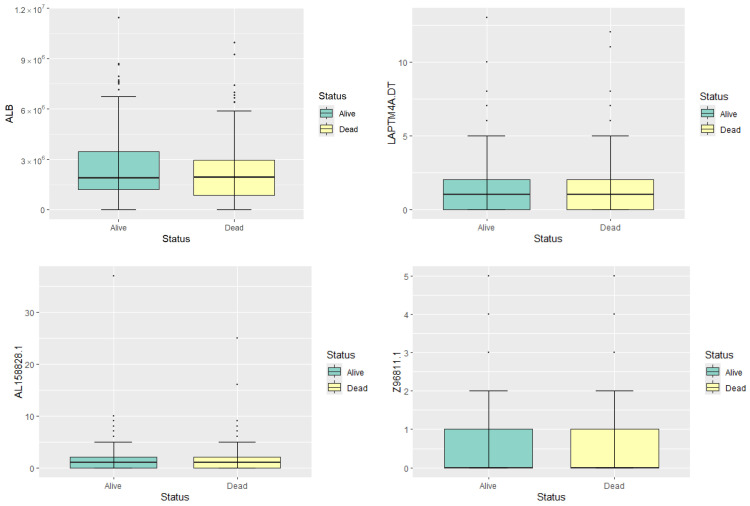
Boxplots of the expression levels of the four genes.

To illustrate the gene expression profiles, we present box plots in Figure 1 and five-number summary statistics in Table 1 for four representative genes: ALB, LAPTM4A.DT, AL158828.1, and Z96811.1. These genes are randomly selected to represent high, moderate, and low expression levels, providing a preliminary overview of gene expression in the dataset. The median expression level of ALB is substantially higher in both survival groups compared to the other genes, indicating its high expression status. Both LAPTM4A.DT and AL158828.1 show moderate levels of expression, with their median and mean values being relatively close. The Z96811.1 gene exhibits the lowest expression levels among the four, with most samples showing very low or zero expression. The gene expression levels of four random selected genes show no significant differences between different statuses. Therefore, feature selection is needed to identify genes with potential prognostic significance.

**Figure 2:**
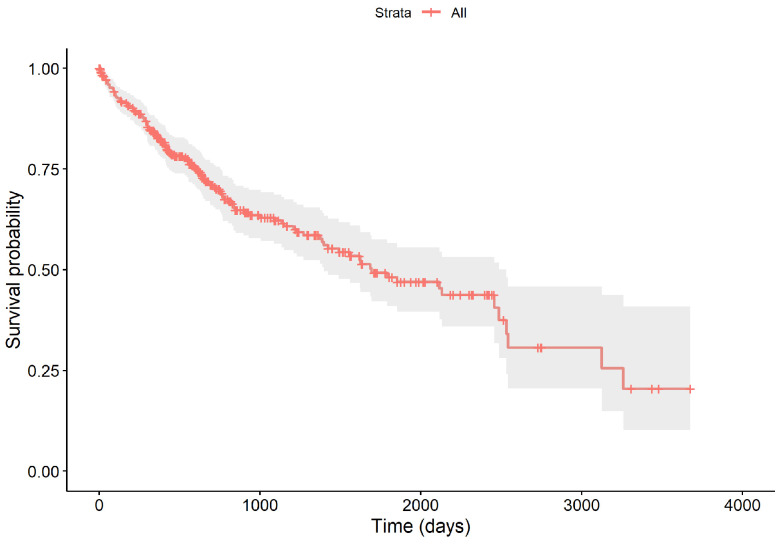
The overall survival curve of 364 LIHC samples.

The survival times of all samples range from 0.02 to 9.53 years. Throughout the follow-up, 130 patients succumbed to the disease, resulting in a censoring rate of 64.28%. The survival curve, depicted in Figure 2, begins at 100% and shows a marked decrease within the first five years, with the survival probability dipping to approximately 48.15% by the end of this period, and a median survival time of 4.62 years. The curve includes vertical drops for deaths, crosses for censoring events, and a gray area indicating the 95% confidence interval of the survival function.

Given the ultra-high dimensionality of the data, where the number of features substantially exceeds the number of observations, special considerations are necessary. Variability in RNA sequencing data often stems from differences in sequencing depth and sample composition, which can skew direct comparisons of expression levels. To address this issue, we employ the Trimmed Mean of M-values (TMM) normalization method as described by Robinson and Oshlack [21]. This method effectively adjusts for inter-sample variations, thereby standardizing gene expression measurements for subsequent analytical assessments. This normalization is crucial for ensuring that comparative analyses of gene expression across samples are both accurate and meaningful.

Survival analysis is a statistical methodology, where researchers systematically track the status of participants over the course of a study until an endpoint, often a terminal event, is reached [14]. This analytical approach primarily focuses on the duration from the onset of individual follow-up to the occurrence of the endpoint. The study employs a hybrid approach integrating the sure independence screening (SIS) method with the Cox proportional hazards model, penalized methods using the least absolute shrinkage and selection operator (Lasso) and smoothly clipped absolute deviation (SCAD), information gain (IG) method, and permutation variable importance (VIMP) for feature selection. The primary objective is to identify significant features that influence the outcome of interest. Following the feature selection phase, we apply three distinct analytical methods, including the Cox model (Cox), survival tree (ST), and random survival forests (RSF), to analyze the data with significant features defined by the results of various feature selection techniques. These methods are utilized to assess the impact of the selected features on the survival outcomes, enabling us to compare the performance of each analytical method across different feature selection results. This comparison helps identify the most effective method for predicting survival, thereby optimizing our analytical strategy.

### 2.2. Cox Proportional Hazards Model

The Cox proportional hazards model is a seminal semi-parametric approach used in the survival analysis. According to Lawless [14], the hazard function at time *t* in relation to covariates $X=(X_{1},X_{2},\ldots ,X_{p})$ is defined as follows:

(1) $$h\left(t,X\right)=h_{0}\left(t\right)\exp\left(X^{T}\beta \right),$$

where $h_{0}\left(t\right)$ denotes the baseline hazard function, representing the hazard when all covariates *X* are set to zero. The component $\exp(X^{T}\beta )$, known as the partial hazard function, accounts for the variation in risk attributable to individual covariate differences.

In this framework, *C* symbolizes the censoring time, and Y=min{t,C} represents the observed event or censoring time. The indicator δ=I(t≤C) determines whether the event is observed (δ=1) or censored (δ=0). Assuming conditional independence between *X* and *Y* given *C*, the observed dataset comprises independent and identically distributed samples $\left\{\left(x_{i},y_{i},\delta_{i}\right):x_{i}\in R^{p},y_{i}\in R^{+},\delta_{i}\in \left\{0,1\right\},i=1,2,\ldots ,n\right\}$. The risk set at time *t*, denoted R(t), includes all individuals for whom $y_{i}\geq t$. The corresponding partial likelihood function is defined as

(2) $$\ell \left(\beta \right)=\ln L\left(\beta \right)=\sum_{i=1}^{n}\delta_{i}x_{i}^{T}\beta -\sum_{i=1}^{n}\delta_{i}\ln\left\{\sum_{j\in R(y_{i})}\exp\left(x_{j}^{T}\beta \right)\right\}.$$

For individuals characterized by covariate vectors *X* and $X^{*}=\left(X_{1}^{*},X_{2}^{*},\ldots ,X_{p}^{*}\right)$, the hazard ratio, representing the relative risk between two individuals, is expressed as

(3) $$\hat{\mathrm{HR}}=\frac{\hat{h}\left(t,X^{*}\right)}{\hat{h}\left(t,X\right)}=\exp\left[\sum_{i=1}^{p}{\hat{\beta }}_{i}\left(X_{i}^{*}-X_{i}\right)\right].$$

This formulation reveals that the baseline hazard functions cancel out, leading to a hazard ratio that remains constant over time, provided the covariates are time-independent.

### 2.3. Feature Selection

In our study focusing on survival outcomes, we face the challenge of handling ultra-high dimensional data, where the number of predictors (*p*) significantly exceeds the number of observations (*n*). This imbalance (p>n) can lead to issues such as overfitting, computational inefficiency, and challenges in model interpretability [6,7]. To effectively address these challenges, we employ the sure independence screening method, specifically designed to reduce the dimensionality from p>n to p<n. This reduction is crucial, as it enables the application of penalized feature selection methods like Lasso, which are otherwise ineffective or unstable in a p>n setting [6,19]. By reducing the number of predictors, SIS not only enhances the feasibility of subsequent analysis but also improves the accuracy and stability of the survival models we develop. Following this dimensionality reduction, we further refine our feature selection process using penalized methods, including Lasso and SCAD, as well as IG and VIMP methods, specifically adapted for the Cox model. These approaches help us to isolate and retain only those features that are most predictive of the survival outcome, thereby enhancing both the interpretability and the predictive accuracy of our method.

#### 2.3.1. Sure Independence Screening

Sure independence screening was originally developed for linear models and later extended to accommodate more complex models, including generalized linear models, robust regression models, and the Cox proportional hazards model [6,7]. Let $M^{*}$ denote the index set of the sparse model, specifically defined as $M^{*}=\left\{j:\beta_{j}^{*}\neq 0,1\leq j\leq p\right\}$, where $\beta_{j}^{*}$ are the true regression coefficients within the Cox model. The efficacy of the feature selection method hinges on the probability that the selected model $\hat{M}$ encompasses the true model $M^{*}$, ideally approaching unity, thereby meeting the criterion of sure screening. Fan and Lv [6] demonstrated that SIS possesses these sure screening properties under certain regularity conditions. For each feature $X_{m}$ with 1≤m≤p, its marginal utility is determined by maximizing the partial likelihood function for that single covariate:

(4) $$u_{m}=\max_{\beta_{m}}\left(\sum_{i=1}^{n}\delta_{i}x_{im}\beta_{m}-\sum_{i=1}^{n}\delta_{i}\log\left\{\sum_{j\in R(y_{i})}\exp\left(x_{jm}\beta_{m}\right)\right\}\right),$$

where $x_{im}$ represents the *m*-th element of $x_{i}$, i.e., $x_{i}={\left(x_{i1},x_{i2},\ldots ,x_{ip}\right)}^{T}$. The marginal utility reflects the amount of survival information that each covariate contributes. After computing the marginal utilities $u_{m}$ for all covariates, they are ranked based on these utilities, and the top *d* covariates are selected, forming the index set *I*. When *d* is chosen to be sufficiently large, the set *I* is highly likely to cover the true model $M^{*}$, ensuring the sure screening property of the SIS approach. However, as *d* increases, the index set *I* may incorporate some irrelevant covariates.

#### 2.3.2. Feature Selection

The objective function for the penalized Cox regression approach is defined by the following expression:

(5) $$Q\left(\beta \right)=\ell \left(\beta \right)-\sum_{j=1}^{p}P_{\lambda }\left(\beta_{j}\right),$$

where ℓ(β) is derived from the log-partial likelihood function referenced as (2). Optimization of this function, achieved through its maximization, facilitates the identification of significant features within the dataset.

The penalized feature selection described by the objective function in Equation (5) employs various regularization penalties, including Lasso and SCAD. Lasso utilizes L1 regularization to encourage sparsity among the coefficient estimates. According to  Tibshirani [19], the Lasso penalty function is defined as

(6) $$P_{\lambda }\left(\beta \right)=\lambda {\left|\beta \right|}_{1}$$

where λ is a regularization parameter controlling the extent of sparsity. SCAD is a nonconvex regularization approach designed to overcome some of the limitations of Lasso, particularly the bias in large coefficient estimates [20]. The SCAD penalty is defined as follows

(7) $$p_{\lambda }\left(\beta \right)=\left\{\begin{array}{cc} \lambda |\beta |, & \mathrm{if}\;|\beta |\leq \lambda \\ \frac{-\left({\left|\beta \right|}^{2}-2a\lambda \left|\beta \right|+\lambda^{2}\right)}{2(a-1)}, & \mathrm{if}\;\lambda <|\beta |\leq a\lambda \\ \frac{\left(a+1\right)\lambda^{2}}{2}, & \mathrm{if}\;|\beta |>a\lambda \end{array}\right.$$

where λ is a regularization parameter that controls the strength of the penalty, influencing the sparsity of the model by shrinking smaller coefficients toward zero. The parameter *a*, typically set at 3.7, controls the transition between the $L_{1}$-type penalty and a constant penalty to reduce bias in large coefficient estimates. The choice of λ and *a* is critical for balancing feature selection with estimation accuracy, ensuring that the SCAD penalty effectively identifies important features while minimizing bias in the estimation of large coefficients.

Information gain is a feature selection method derived from information theory that measures the reduction in entropy or uncertainty about the target variable given a particular feature [12]. For a feature $X_{j}$, the information gain $IG(X_{j})$ is defined as

(8) $$IG\left(X_{j}\right)=H\left(Y\right)-H\left(Y|X_{j}\right),$$

where H(Y) is the entropy of the target variable *Y*, and $H(Y|X_{j})$ is the conditional entropy of *Y* given the feature $X_{j}$. The entropy *H* is calculated as

(9) $$H\left(Y\right)=-\sum_{k=1}^{K}P\left(y_{k}\right)\log P\left(y_{k}\right),$$

where $P(y_{k})$ is the probability of the target variable *Y* taking the value $y_{k}$. By selecting features that provide the highest information gain, we can identify those that most effectively reduce uncertainty about the target variable.

In addition to the aforementioned feature selection methods, we incorporate permutation variable importance (VIMP) provided by random survival forests [18,22]. VIMP is a measure of the significance of a variable in predicting the outcome by quantifying the increase in prediction error when the values of that variable are randomly permuted. To compute VIMP, the RSF model is trained on a bootstrap sample, and the prediction error is calculated using out-of-bag (OOB) data. The values of a specific variable are then permuted in the OOB data, and the prediction error is recalculated. The difference in prediction error before and after permutation represents the importance of that variable. A higher VIMP score indicates a more significant impact on the prediction accuracy, highlighting the variable’s relevance in the model.

### 2.4. Survival Tree

The survival tree method, an extension of the classification and regression tree methodology, is specifically designed to accommodate censored survival data [16]. The ST constructs a binary tree through recursive partitioning, initiating with the root node. Each node split is determined based on a survival criterion that maximizes the differences in survival distributions between child nodes. This is formally represented as

(10) $$G\left(s^{*},h\right)=\max_{s\in S_{h}}G\left(s,h\right),$$

where G(s,h) denotes the logrank test statistic for the set of samples at node *h*, *s* encompasses all feasible splits within the node, and $s^{*}$ signifies the optimal split. This recursive partitioning continues until a predefined stopping criterion is met, effectively segmenting the data into groups with significantly different survival experiences.

The output of the survival tree method is presented in a tree structure. This format is not only intuitive and straightforward but also facilitates an easy comprehension and communication of the results. Thus, the survival tree serves as a robust tool for exploring complex interactions and nonlinear relationships in survival data.

### 2.5. Random Survival Forests

Random survival forests is an extension of the random forest method designed to handle right-censored survival data through the construction of survival trees and an ensemble cumulative hazard function [18,23]. The RSF method operates as follows:

1. Bootstrap Sampling: Extract *B* bootstrap samples from the original dataset, with each sample typically containing about 63% of the total sample size. The samples not included in a bootstrap are termed out-of-bag data.
2. Survival Tree Construction: For each bootstrap sample, a binary survival tree is constructed. At each node, *p* features are randomly selected, and the best split is chosen based on the maximum survival difference between the resultant child nodes.
3. Tree Growth: Tree development continues, ensuring that the number of samples at each leaf node remains above a minimum threshold $d_{0}>0$.
4. Cumulative Hazard Function: Compute the cumulative hazard function for each tree and average these functions across the forest to produce an ensemble cumulative hazard function.
5. Error Estimation: Utilize the OOB data to assess the prediction error of the ensemble cumulative hazard function.

During tree construction, the splitting criteria must utilize survival time and censoring data, employing survival differences to assess node purity and the effectiveness of splits.

### 2.6. Performance Metrics

Our study utilizes several sophisticated metrics to assess the performance of survival methods, including the time-dependent receiver operating characteristic (ROC) curve, area under the curve (AUC), concordance Index (C-index), specificity, sensitivity, negative predictive value (NPV), and positive predictive value (PPV). The time-dependent ROC, adapted for survival analysis, evaluates the method accuracy at various time points by calculating sensitivity and specificity.

At a given time point *t*, specificity is defined as

(11) $$\mathrm{Specificity}\left(t\right)=\frac{\mathrm{TN}(t)}{\mathrm{FP}(t)+\mathrm{TN}(t)},$$

where TN(t) denotes the number of individuals correctly predicted as not having the event occur at or after time *t*, and FP(t) represents the individuals who, at or after time *t*, did not have the event but were incorrectly predicted to have it. Sensitivity, also known as the true positive rate, measures the proportion of correctly identified cases where an event, such as death or disease recurrence, has occurred. The formula for sensitivity at a given time point *t* is

(12) $$\mathrm{Sensitivity}\left(t\right)=\frac{\mathrm{TP}(t)}{\mathrm{FN}(t)+\mathrm{TP}(t)},$$

where TP(t) denotes the number of individuals correctly predicted to have the event occur at or before time *t*, and FN(t) represents those who had the event occur at or before time *t* but were not correctly predicted by the model. Negative predictive value measures the proportion of true negatives among all individuals predicted as negative, and its formula at a given time point *t* is

(13) $$\mathrm{NPV}\left(t\right)=\frac{\mathrm{TN}(t)}{\mathrm{TN}(t)+\mathrm{FN}(t)}.$$

The positive predictive value measures the proportion of true positives among all individuals predicted as positive, and its formula at a given time point *t* is

(14) $$\mathrm{PPV}\left(t\right)=\frac{\mathrm{TP}(t)}{\mathrm{TP}(t)+\mathrm{FP}(t)}.$$

These measures facilitate the construction of the ROC curve, from which the AUC is derived to quantify the method’s overall predictive performance. To plot the time-dependent ROC curve and calculate the AUC value at a follow-up time of one year, we utilize the timeROC library in R, which provides tools tailored for handling censored data in survival analysis [24]. This approach ensures that the ROC curve and AUC calculations account for the unique aspects of survival data, thereby providing a more robust evaluation of model performance. Furthermore, the C-index is employed to evaluate the concordance between predicted outcomes and actual events. It is defined as

(15) $$C_{\mathrm{index}}=\frac{\sum_{i,j\in \Omega }\left(I\left\{{\hat{s}}_{i}<{\hat{s}}_{j}\right\}+0.5\times I\left\{{\hat{s}}_{i}={\hat{s}}_{j}\right\}\right)}{\left|\Omega \right|},$$

where *I* is the indicator function, and Ω is the set of all patient pairs {i,j} that are comparable. Here, ${\hat{s}}_{i}$ and ${\hat{s}}_{j}$ represent the estimated survival probabilities for patients *i* and *j*, respectively. This index critically assesses the discriminative power of the method, particularly its capability to accurately rank patients according to their survival times. These comprehensive evaluations underscore the methods’ effectiveness in clinical research settings.

## 3. Results

### 3.1. Identification of Genetic Markers

In our investigation of ultra-high-dimensional LIHC RNA-seq data, we initiated our analysis by employing the sure independence screening (SIS) method [6,7]. This initial step aimed to streamline the dataset by reducing the number of features to a manageable size, specifically to 62 gene features. This approach adheres to the guidelines proposed by Fan and Lv [6], recommending the parameter setting $d=\frac{n}{\ln(n)}$, where *d* denotes the number of features retained, and n=364 is the sample size of our study. To assess the effectiveness of this dimension reduction, we conducted an exploratory analysis to estimate the variance retained by comparing the total variance before and after applying SIS. Our analysis reveals that approximately 85.465% of the total variance is retained. This indicates that, despite the significant reduction in the number of genes, a substantial portion of the original data’s variance, and consequently much of the underlying biological information, is preserved. The retained variance suggests that the selected genes still capture the majority of the key signals necessary for accurate prognostic predictions, validating the effectiveness of our dimension reduction approach. Following the initial dimension reduction, we apply feature selection methods, including penalized methods with Lasso and SCAD, IG, and VIMP, to perform more refined feature selection [12,19,20,22]. These feature selection methods, combined with SIS, are respectively referred to as SIS-Lasso, SIS-SCAD, SIS-IG, and SIS-VIMP.

Table 2 presents the features selected by the SIS-Lasso, SIS-SCAD, SIS-IG, and SIS-VIMP methods. Each method identifies a unique set of features, with some overlap among them, reflecting their distinct approaches to penalization and selection criteria. The SIS-Lasso method selects a total of 11 features. Lasso’s L1 regularization tends to produce sparse models by driving some coefficients to zero, which is evident in its selection of features. The SIS-SCAD method selects six features. The SCAD penalty, designed to reduce the bias in large coefficient estimates, leads to the selection of a more refined set of features. Notably, all features selected by SIS-SCAD are also included in the features selected by SIS-Lasso. The SIS-IG method identifies nine features, and the SIS-VIMP method also identifies nine features. Information gain, derived from information theory, measures the reduction in entropy provided by each feature. Interestingly, SIS-IG has the least overlap with the other two methods, sharing only two features (MED8 and CLEC3B) with SIS-Lasso and only one feature (MED8) with SIS-SCAD. SIS-VIMP, which uses variable importance scores from random survival forests, shares four features (SLC41A3, YARS1, MED8, and CCDC58) with SIS-Lasso and three features (SLC41A3, YARS1, and MED8) with SIS-SCAD. This indicates that SIS-IG and SIS-VIMP may capture different aspects of the data, potentially identifying features that contribute uniquely to the prediction model.

**Figure 3:**
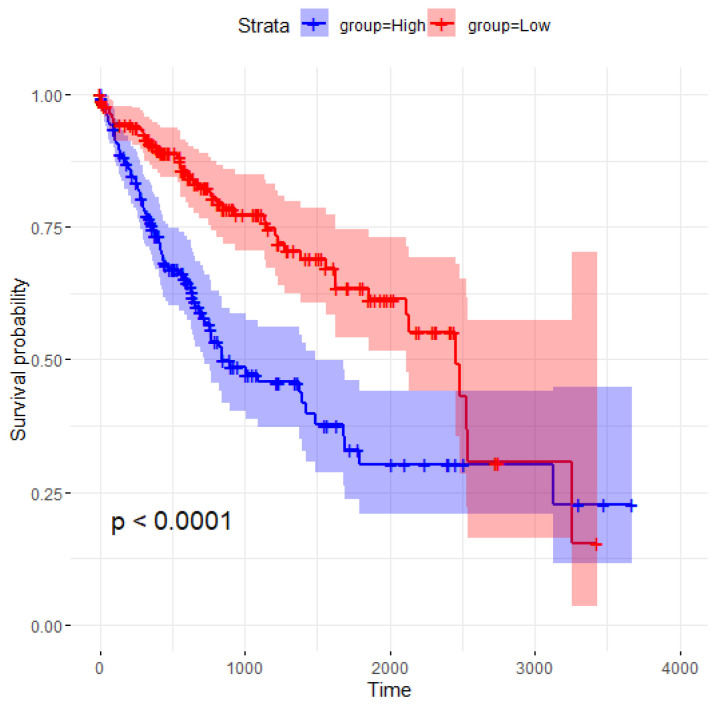
Kaplan–Meier survival curve for MED8 samples.

A closer look at the overlapping features reveals that MED8 is consistently selected by all four methods, underscoring its importance. The Kaplan–Meier survival curve for MED8 and the log-rank test *p*-value are shown in Figure 3, demonstrating a significant difference in survival probabilities between the high- and low-expression groups. The division into “High” and “Low” expression groups is based on the median value of MED8, which is calculated to be 7534.931. Samples with MED8 expression values below the median are categorized as “Low”, while those with values at or above the median are categorized as “High”. The high-expression group (blue curve) exhibits markedly lower survival probabilities over time compared to the low-expression group (red curve). The log-rank test yields a *p*-value of less than 0.0001, indicating that the difference in survival between the two groups is statistically significant. These results suggest that MED8 expression levels are strongly associated with patient survival outcomes, with higher expression correlating with poorer prognosis.

Also, as shown in Table 2, CAD, SLC41A3, YARS1, GNL2, and KPNA2 are chosen by both SIS-Lasso and SIS-SCAD, suggesting that these features are robustly informative across different penalization schemes. The SIS-VIMP method further reinforces the importance of SLC41A3, YARS1, MED8, CCDC58, and KPNA2, which are also selected by SIS-Lasso and SIS-SCAD. Conversely, features like POLR3G, PLEKHA8P1, and CAPN10 are uniquely selected by SIS-Lasso, while RBM28, TRMT6, NUP107, DDX55, RUVBL1, METTL3, and DHX37 are unique to SIS-IG, highlighting method-specific insights. The SIS-VIMP method also identifies unique features such as AP000439.2, CDKL3, and NSA2P7, which are not selected by any of the other methods. These results underscore the complementary nature of different feature selection methods. While some features are universally recognized as important, others are method specific, offering a more diverse perspective on the underlying data structure. The relatively lower overlap between SIS-IG and the other three methods suggests that information gain may provide unique insights not captured by Lasso, SCAD, or VIMP penalizations. The unique selections by SIS-IG and SIS-VIMP could be attributed to their ability to capture features that significantly reduce the entropy or enhance the model’s predictive accuracy by incorporating a broader range of informative features. The diversity in feature selection across these methods highlights the importance of considering multiple approaches to capture the most comprehensive set of informative features for the predictive model.

**Figure 4:**
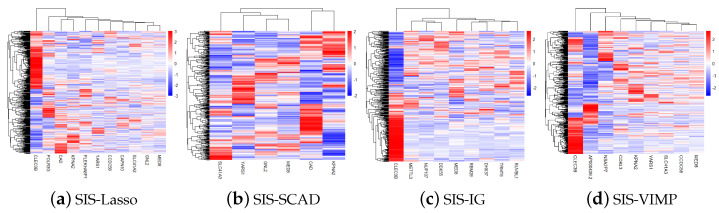
Gene heatmaps of features selected by sure independence screening coupled with Lasso (SIS-Lasso) (**a**), smoothly clipped absolute deviation (SIS-SCAD) (**b**), information gain (SIS-IG) (**c**), and permutation variable importance provided by random survival forests (SIS-VIMP) (**d**). These heatmaps illustrate the distinct gene expression patterns identified by each feature selection method, highlighting the diversity and potential complementary nature of the selected features across different methodologies.

Figure 4 shows the gene heatmaps for the features selected by the SIS-Lasso method, SIS-SCAD, and SIS-IG. The gene heatmaps reveal distinct clustering patterns for each feature selection method. SIS-Lasso selects a broad set of features with varied expression levels, while SIS-SCAD focuses on a subset of these features with more defined expression clusters. SIS-IG highlights unique features with minimal overlap with the other methods, suggesting different expression profiles. The SIS-VIMP heatmap showcases features selected based on their importance scores, further emphasizing distinct clustering patterns that may not be captured by the other methods. These heatmaps illustrate the diverse gene expression patterns captured by each selection technique.

**Figure 5:**
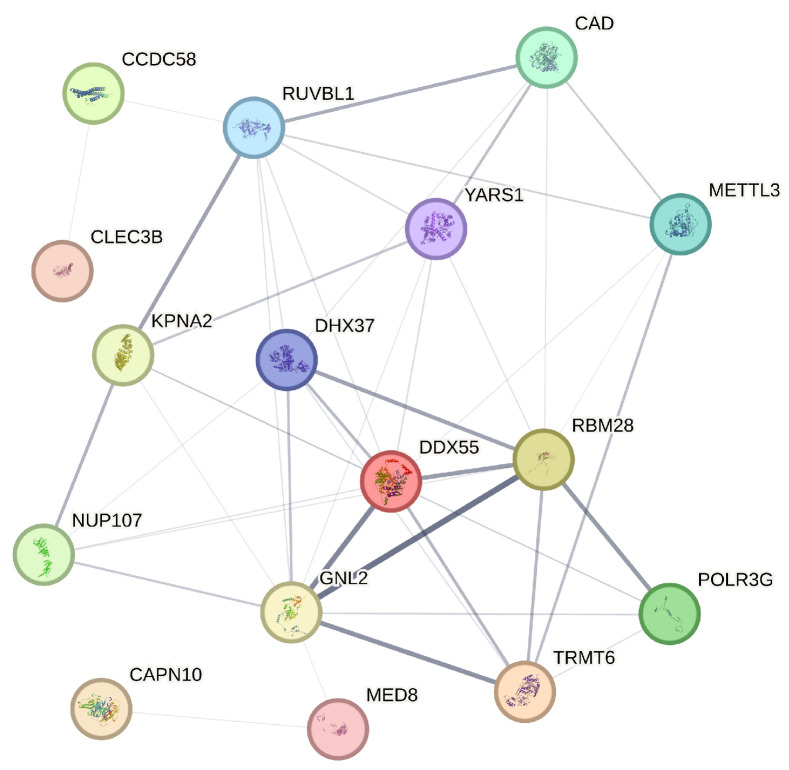
Protein–protein interaction network illustrating the interactions among 16 selected genes.

The STRING database is a comprehensive resource that aggregates protein–protein interaction data from various sources to construct interaction networks, thereby providing insights into the biological relationships between proteins [25]. In our analysis, we utilize STRING to generate a protein interaction network for the 21 genes identified through our four variable selection methods. STRING assigns a confidence score to each interaction, derived from a combination of evidence sources, reflecting the strength and biological relevance of the interaction. In constructing our network, we apply a confidence threshold of 0.15 to ensure that only the most reliable interactions are included. As a result of this threshold, genes such as NSA2P7, AP000439.2, and PLEKHA8P1, which do not meet the minimum interaction data requirements, are excluded from the final network diagram. The refined network, as shown in Figure 5, encompasses 16 genes, each represented as a node, with the thickness of the connecting edges corresponding to the strength of the interaction evidence between the encoded proteins.

The analysis of the network diagram reveals several key insights. Firstly, genes like RUVBL1 and DDX55, which occupy central positions with numerous connections, appear to be pivotal in the protein interaction network of liver hepatocellular carcinoma (LIHC), suggesting their potential as key regulatory hubs in LIHC-related processes. Additionally, the presence of functional clusters, such as the interactions among KPNA2, DHX37, and YARS1, indicates possible shared roles in critical cellular processes like RNA processing, which are often implicated in cancer progression. The absence of certain genes due to insufficient interaction data points to areas that may require further exploration, as these genes could be involved in less characterized pathways. Furthermore, the variation in edge thicknesses among connections highlights the differing levels of evidence for each interaction, with genes like MED8 and GNL2 showing strong interactions that suggest well-established roles in LIHC mechanisms. These findings align with the study’s broader objective of identifying actionable prognostic markers and underscore the value of integrating network-based analysis with gene selection methods to enhance our understanding of complex diseases like LIHC.

### 3.2. Survival Analysis

After conducting feature selection using SIS combined with Lasso, SCAD, and information gain, we employ three survival analysis methods, including the Cox proportional hazard model, survival tree, and random survival forests, to analyze the data with the selected features. We evaluate the effectiveness of each survival analysis method using a 10-fold cross-validation approach. In this method, the genetic data are randomly divided into ten subsets. Each time, nine subsets (90%) are used for training, and the remaining one subset (10%) is used for testing. This process is repeated ten times to ensure robust validation. Methods are fit on the training set and their performance are assessed on the testing set using the receiver operating characteristic curve, area under the curve, concordance index, sensitivity, specificity, negative predictive value, and positive predictive value.

**Figure 6:**
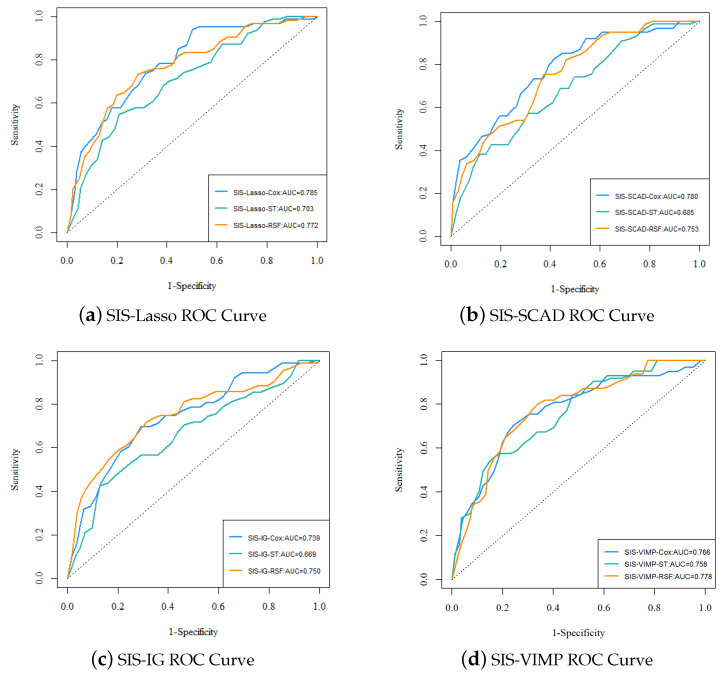
The ROC curves and AUC values for each analysis method with different feature selection approaches. (**a**) ROC curve for SIS-Lasso, showing the performance of Cox, ST, and RSF methods; (**b**) ROC curve for SIS-SCAD, displaying the performance of Cox, ST, and RSF methods; (**c**) ROC curve for SIS-IG, highlighting the performance of Cox, ST, and RSF methods; (**d**) ROC curve for SIS-VIMP, demonstrating the performance of Cox, ST, and RSF methods.

Figure 6 presents the ROC curves for the different analysis methods (Cox, ST, RSF) with various feature selection approaches (SIS-Lasso, SIS-SCAD, SIS-IG, and SIS-VIMP). The ROC curves provide a visual comparison of the predictive performance across different methods and feature selection techniques. The performance of SIS-Lasso consistently shows strong predictive capability across all methods, particularly with the Cox and RSF methods. SIS-SCAD also performs well, closely following SIS-Lasso, while SIS-IG, despite capturing unique aspects of the data, exhibits slightly less effective performance. The SIS-VIMP method demonstrates competitive performance, particularly with the RSF method, highlighting its potential as a valuable feature selection approach. These results emphasize the importance of selecting an appropriate feature selection method to enhance the performance of survival analysis methods.

The performance metrics for each analysis method with different feature selection approaches are summarized in Table 3. From the ROC curves in Figure 6 and the performance metrics in Table 3, it is evident that SIS-Lasso consistently demonstrates strong performance across all analysis methods. The C-index values further corroborate this, showing that SIS-Lasso achieves high predictive accuracy with the Cox and RSF methods, specifically with C-index values of 0.693 and 0.704, respectively. SIS-SCAD also performs well, closely following SIS-Lasso, particularly with the Cox method. SIS-IG, while still effective, shows slightly lower performance metrics across the methods. The SIS-VIMP approach demonstrates competitive performance, particularly with the RSF method, where it achieves the highest C-index value of 0.720. The sensitivity and specificity metrics reveal that SIS-Lasso performs effectively in identifying true positive cases, with sensitivity values around 0.834, while SIS-VIMP shows promising results with higher specificity and sensitivity values compared to the other methods. The NPV and PPV values suggest that while SIS-Lasso has high reliability in negative predictions (NPV around 0.927), SIS-VIMP offers improved balance between positive and negative prediction reliability, with a higher NPV (0.960) and slightly better PPV (0.278) than the other methods.

**Figure 7:**
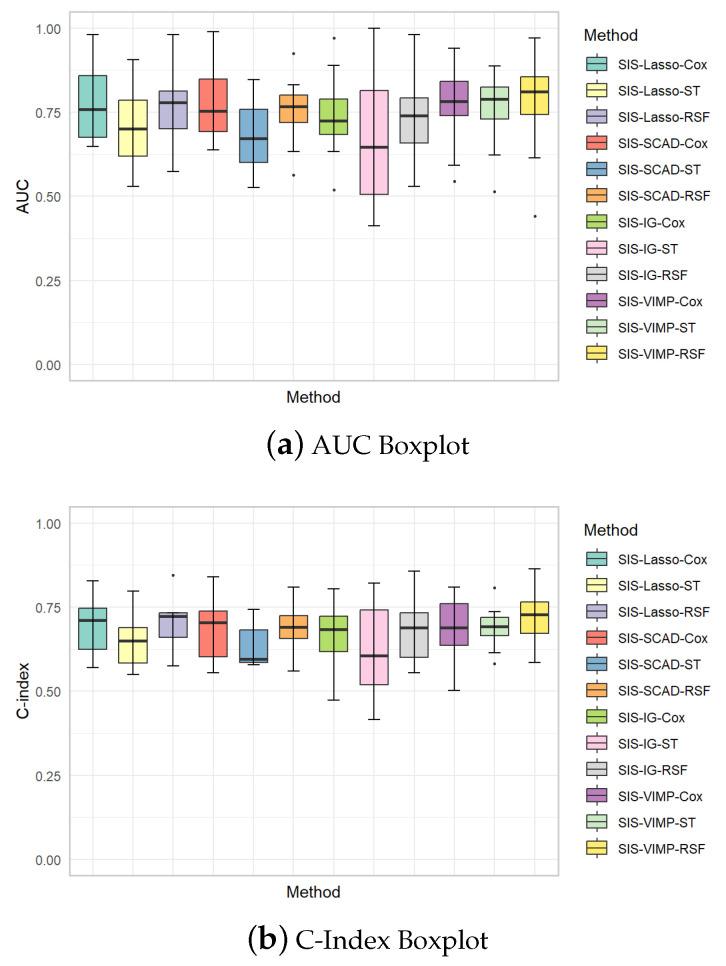
Boxplots of AUC and C-index values for each analysis method with different feature selection approaches. (**a**) AUC Boxplot: Displays the area under the curve (AUC) for various methods to evaluate the overall performance. (**b**) C-Index Boxplot: Shows the concordance index (C-index) which measures the discriminatory power of the models in predicting the time-to-event data.

The boxplots in Figure 7 provide additional insights into the variability of the AUC and C-index values across different methods. These plots illustrate the distribution of performance metrics for each method, showing the median, interquartile range, and potential outliers. The AUC boxplots reveal that SIS-Lasso and SIS-VIMP generally achieve the highest median AUC values, with SIS-Lasso showing relatively low variability across the different methods, indicating consistent performance. SIS-SCAD and SIS-IG, however, display greater variability, especially for the ST model, which suggests less stable performance. Similarly, the C-index boxplots indicate that SIS-Lasso and SIS-VIMP consistently achieve higher median C-index values compared to SIS-SCAD and SIS-IG. While the C-index values for SIS-SCAD exhibit moderate variability, SIS-IG shows the widest range, reflecting considerable variability in its predictive accuracy. These boxplots underscore the robustness of SIS-Lasso and SIS-VIMP in providing reliable and stable model performance, particularly with the Cox and RSF methods.

## 4. Discussion and Conclusions

In this study, we explored the genetic underpinnings of liver hepatocellular carcinoma (LIHC) using RNA sequencing data from the Cancer Genome Atlas (TCGA). Our objective was to identify critical genetic markers that could serve as potential prognostic indicators and to compare the effectiveness of various feature selection and survival analysis methods in handling ultra-high-dimensional data.

We employed a multi-step approach to feature selection, beginning with the sure independence screening (SIS) method to reduce the dimensionality of the dataset, followed by the application of penalized methods such as Lasso and SCAD, and the information gain method. To further refine the selection process, we incorporated permutation variable importance (VIMP) from random survival forests (RSF). This comprehensive feature selection strategy enabled us to identify a diverse set of significant genes, each contributing uniquely to the survival outcomes of LIHC patients.

The features selected by the SIS-Lasso, SIS-SCAD, SIS-IG, and SIS-VIMP methods highlighted the complementary nature of different selection techniques. While some features like MED8 were consistently identified across all methods, others were method specific, underscoring the importance of employing multiple approaches to capture the full spectrum of informative features. This diversity in feature selection suggests that different methods can provide unique insights into the underlying data structure, which is critical for developing robust prognostic models. The identification of MED8 as a consistently significant feature across multiple selection methods, coupled with its strong association with survival outcomes in LIHC patients, underscores its potential as a valuable prognostic biomarker. Our findings align with the research conducted by Jin et al. [26], which demonstrated that MED8 is overexpressed in hepatocellular carcinoma tissues and that higher levels of MED8 correlate with poorer outcomes in LIHC. Jin et al. [26] further confirmed the critical role of MED8 in tumor biology by showing that the gene knockdown of MED8 led to a reduction in both the proliferation and invasiveness of liver cancer cells. This evidence supports the broader adoption of MED8 as a biomarker for LIHC prognosis and possibly as a therapeutic target. Similarly, SLC41A3 emerged as an important feature across multiple selection methods in our study, reinforcing its potential as a prognostic marker. The existing literature supports this finding, with studies by Chang et al. [27] and Li et al. [28] indicating that the high expression of SLC41A3 predicts poor prognosis in LIHC patients. These studies suggest that SLC41A3 might be a promising target for LIHC treatment, further validating its significance in our analysis. Other features, such as YARS1 and KPNA2, also emerged as potentially valuable biomarkers or therapeutic targets, although further research is needed to explore their roles in LIHC progression.

Our comprehensive analysis demonstrated that the random survival forests method consistently outperformed traditional statistical approaches, including the Cox proportional hazards model and survival trees, especially when coupled with the SIS-Lasso and SIS-VIMP feature selection methods. The performance metrics, such as ROC curves, C-index, sensitivity, and specificity, consistently indicated that RSF, in combination with these advanced feature selection techniques, offered superior predictive accuracy, robustness, and stability. This underscores the exceptional capability of RSF to manage the intricacies of complex, high-dimensional datasets like RNA sequencing data, where traditional methods might struggle. The inherent flexibility of RSF allows it to effectively capture nonlinear relationships and interactions among features, making it particularly advantageous in scenarios where data complexity and feature interdependencies are prominent. Therefore, RSF, combined with SIS-Lasso or SIS-VIMP, should be considered a powerful tool for researchers dealing with high-dimensional genomic data, where precise survival predictions are crucial for advancing personalized medicine and targeted treatment strategies.

Our findings highlight the crucial role of integrating advanced machine learning techniques with traditional statistical methods to enhance the predictive accuracy of survival analysis in high-dimensional genomic data. The hybrid approach combining SIS with penalized regression methods, information gain, and VIMP not only improves feature selection but also deepens our understanding of the genetic mechanisms underlying LIHC. This integrated methodology is particularly valuable in identifying significant prognostic markers that could be leveraged in clinical settings. For example, the robust identification of markers like MED8 underscores their potential not only as diagnostic tools but also as targets for therapeutic intervention. Ultimately, this study provides a solid framework for the analysis of high-dimensional genomic data, demonstrating how combining advanced machine learning techniques with traditional statistical methods can lead to the identification of actionable prognostic markers. These markers, once validated in clinical trials, have the potential to transform patient care by enabling more precise risk stratification and the development of tailored treatment regimens. By facilitating the early identification of high-risk patients and guiding the selection of targeted therapies, this approach could significantly improve patient outcomes, reduce treatment-related morbidity, and optimize resource allocation in clinical settings. The integration of these findings into routine clinical practice represents a critical step forward in the realization of personalized medicine in oncology, particularly for liver cancer.

Despite these promising results, there are limitations in our study. The exclusion of clinical features may overlook important interactions between genetic and clinical factors. Future research should integrate clinical features to provide a more comprehensive model. Additionally, validation with larger, independent datasets is necessary to generalize the findings. Further exploration into the biological mechanisms underlying the identified genetic markers could offer deeper insights into LIHC pathogenesis and potential therapeutic targets. Furthermore, future research could also consider stratifying the sample by progression stage to enhance model accuracy, particularly in studies with larger sample sizes, where stratification could reveal stage-specific genetic features and improve the robustness of the model.

## Figures and Tables

**Table 1:** Summary statistics of the four genes. The table presents the minimum (Min.), first quartile (1st Qu.), median, mean, third quartile (3rd Qu.), and maximum (Max.) expression values for each gene.

	ALB	LAPTM4A.DT	AL158828.1	Z96811.1
Min.	1079	0.000	0.000	0.000
1st Qu.	1,122,048	0.000	0.000	0.000
Median	1,936,114	1.000	1.000	0.000
Mean	2,420,186	1.489	1.489	0.544
3rd Qu.	3,195,438	2.000	2.000	1.000
Max.	11,448,806	13.000	37.000	5.000

**Table 2:** Selected features by SIS-Lasso, SIS-SCAD, SIS-IG, and SIS-VIMP.

Feature	SIS-Lasso	SIS-SCAD	SIS-IG	SIS-VIMP
CAD	✓	✓		
POLR3G	✓			
SLC41A3	✓	✓		✓
PLEKHA8P1	✓			
YARS1	✓	✓		✓
GNL2	✓	✓		
CAPN10	✓			
MED8	✓	✓	✓	✓
CCDC58	✓			✓
CLEC3B	✓		✓	✓
KPNA2	✓	✓		✓
RBM28			✓	
TRMT6			✓	
NUP107			✓	
DDX55			✓	
RUVBL1			✓	
METTL3			✓	
DHX37			✓	
AP000439.2				✓
CDKL3				✓
NSA2P7				✓
**Count**	11	6	9	9

**Table 3:** Performance metrics for each analysis method with different feature selection approaches.

Metrics	SIS-Lasso			SIS-SCAD			SIS-IG			SIS-VIMP		
	**Cox**	**ST**	**RSF**	**Cox**	**ST**	**RSF**	**Cox**	**ST**	**RSF**	**Cox**	**ST**	**RSF**
AUC	0.785	0.703	0.772	0.780	0.685	0.753	0.739	0.669	0.750	0.766	0.758	0.778
C-index	0.693	0.652	0.704	0.686	0.631	0.685	0.669	0.623	0.682	0.684	0.688	0.720
Sensitivity	0.834	0.843	0.833	0.827	0.643	0.780	0.816	0.707	0.863	0.802	0.800	0.843
Specificity	0.458	0.370	0.441	0.429	0.582	0.462	0.412	0.440	0.391	0544	0.414	0.467
NPV	0.927	0.908	0.927	0.931	0.906	0.933	0.929	0.910	0.925	0.936	0.935	0.960
PPV	0.260	0.212	0.250	0.249	0.249	0.247	0.226	0.200	0.244	0.278	0.236	0.263

## Data Availability

The data presented in the study are available in the Cancer Genome Atlas database at https://portal.gdc.cancer.gov, accessed on 12 February 2024.

## Abbreviations

The following abbreviations are used in this manuscript:

LIHC	Liver Hepatocellular Carcinoma
TCGA	The Cancer Genome Atlas
GDC	Genomic Data Commons
SIS	Sure Independence Screening
Lasso	Least Absolute Shrinkage and Selection Operator
SCAD	Smoothly Clipped Absolute Deviation
IG	Information Gain
ST	Survival Tree
RSF	Random Survival Forests
ROC	Receiver Operating Characteristic
AUC	Area Under the Curve
C-index	Concordance Index
TMM	Trimmed Mean of M-values
